# Don’t sell out safety: a call to preserve risk evaluation and mitigation strategies to reduce harm to patients and the public in the U.S.

**DOI:** 10.1186/s40545-016-0051-0

**Published:** 2016-01-21

**Authors:** Stacey L. Worthy

**Affiliations:** Alliance for the Adoption of Innovations in Medicine (Aimed Alliance), 1000 Potomac St. NW, Suite 150-A, Washington, DC 20007 USA

**Keywords:** FDA, Risk evaluation and mitigation strategies, REMS, Restricted distribution, Safety, Drugs, Pharmaceuticals

## Abstract

**Background:**

As medicines are becoming more targeted and complex in the U.S., ensuring patients’ safe use of medications with known dangerous risks is critical for public health and safety. Therefore, the Risk Evaluation and Mitigation Strategies (REMS) program is more important than ever. The REMS programs mandates that manufacturers utilize tools to manage known or potential serious risks (e.g., death, severe birth defects, prolonged hospitalization) associated with certain drugs while still making these medications available to patients with unmet medical needs. Yet, recently federal policy makers have proposed legislation to force manufacturers to sell medications with known serious risks in a manner that weakens the medications’ REMS programs.

**Methods:**

The author reviewed U.S. legislation, statutes, case law, government agency policies and guidelines, scholarly articles, and news stories published between January 1, 2004 and December 1, 2015 and provided legal and policy analysis.

**Results:**

REMS are necessary to make medications with known severe risks available to certain patient populations for whom treatment may not be available otherwise.

**Conclusion:**

In order to ensure that proper safety measures are preserved and medications with known risks are not diverted to parties who will not follow safety requirements, legislation should not be passed to require a forced sale of drugs subject to REMS with restricted distribution for bioequivalence testing purposes. Generic manufacturers must be held to the same REMS safety standards as brand manufacturers. Systems currently in place adequately balance risk and safety.

## Introduction

Over the years, certain medicines approved by the U.S. Food and Drug Administration (FDA) have caused significant adverse events once they entered the market, resulting in the need for a regulatory response [1]. One of the best known examples is rofecoxib (Vioxx), the nonsteroidal anti-inflammatory drug (NSAID) that treated adults with arthritis and other painful conditions. FDA approved the medication in May 1999 because it allegedly caused fewer ulcers and less gastrointestinal bleeding than its competitor, naproxen [2]. However, as early as November 1999, a study of 4,000 patients reported that 79 patients taking rofecoxib either died or developed serious heart problems, compared with 41 patients taking naproxen [3]. By the time the manufacturer voluntarily withdrew rofecoxib from the market in 2004, the drug had contributed to more than 60,000 heart attack and stroke-related deaths [4].

With situations like the one surrounding rofecoxib as the impetus, Congress passed the Food and Drug Administration Amendments Act of 2007 (FDAAA), expanding FDA’s authority to regulate the nation’s drug safety system. Taking effect on March 25, 2008, the FDAAA gave FDA broad powers to control drug marketing and labeling, require post-approval studies, establish active surveillance systems, and make clinical trial operations and results more visible to the public [5].

One notable component of the FDAAA was the Risk Evaluation and Mitigation Strategy (REMS) initiative, which provided a new avenue for FDA to approve drugs and biologics that may otherwise have been held up indefinitely due to potential safety concerns [5]. Defined as a “strategy to manage a known or potential serious risk associated with a drug or biological product,” REMS is a mandatory plan that includes risk minimization strategies beyond standard approved labeling to ensure the benefits of a medication outweigh its risks [6]. REMS includes, among other components, medication safety guides, patient package inserts, communications plans, Elements To Assure Safe Use (ETASU), and implementation systems used to monitor, evaluate, and improve application of ETASU [5]. ETASU is the strictest category of REMS and may include restricted distribution systems, which ensure only specifically approved parties have access to the drug under strictly controlled conditions [5].

According to FDAAA, medicines carrying serious risks would be removed from the market altogether without ETASU, leaving certain patient populations without treatment. Yet, critics erroneously argue that brand manufacturers have inappropriately used REMS with ETASU to limit generic manufacturers’ access to quantities of innovator drugs to support bioequivalence testing, a preliminary step in generic drug development [7]. Consequently, Congress has contemplated legislation on several occasions that would force the sale of brand medicines approved with rigorous REMS safeguards to generic manufacturers for bioequivalence testing without sufficient controls to prevent harmful exposure. Although such legislation has failed in the past, it is again under consideration by the 114th Congress.

At a time when medicines are becoming more targeted and complex, ensuring patients’ safe use of medications with known dangerous risks is critical for public health and safety. Therefore, it is vital that policy makers guard closely the drug safety protections REMS make possible while allowing brand and generic manufacturers to negotiate the sale of samples for bioequivalence testing. To document why the current system should not be weakened, this article provides an up-to-date analysis of the REMS program, including its history, the REMS approval process, and enforcement mechanisms. The article then discusses new challenges to REMS that may create unjustified risks to patient safety. It concludes by making recommendations to preserve ETASU, and restricted distribution in particular, as a component of REMS.

## Background

### Overview of the REMS program

Prescription drug safety is a significant concern for millions of Americans who take medications to treat and manage their diseases or disorders [1]. Under the Food, Drug, and Cosmetics Act (FDCA), FDA is responsible for ensuring that all prescription medications are both safe and effective for patient use [1]. Accordingly, FDA’s safety decisions are grounded in the individual context of each drug, such as the nature and severity of the disease that the medication treats and the availability of alternative treatments [1]. In some instances, the only medications available to treat certain disorders may have serious inherent risks or cause severe adverse events, such as birth defects or organ damage, when not handled and administered with utmost care [6]. In these situations, FDA must determine whether the benefit of the medication outweighs the risk to the patient [5].

With the enactment of FDAAA in 2007, Congress expanded FDA’s powers to assure the safety of prescription medicines. This included new authority to require REMS to manage known or potential serious risks associated with certain drugs while still making these medications available to patients with unmet medical needs [1, 8]. The REMS program allows FDA to approve a drug that would otherwise be denied approval due to safety issues [9]. Therefore, FDA’s expanded authority is meant to strengthen its ability to safeguard the public, and it gives the agency options when serious risks are discovered after a drug has been approved and brought to market [10].

FDA determines whether a REMS is required either during or after the approval process [11]. In evaluating the necessity of REMS, FDA will consider the following elements:

- Whether the drug is a new molecular entity;
- Seriousness of the disease or condition that the drug treats;
- Expected benefit of the drug;
- Expected or actual duration of treatment;
- Estimated size of the population likely to use the drug;
- Seriousness of any known or potential adverse events related to the drug; and
- History of such incidences [5].

If FDA decides the risk-benefit profile warrants a REMS, the agency will require the manufacturer to submit a REMS proposal containing goals, elements, tools, and an assessment plan [5]. Following implementation of the REMS plan, the manufacturer must submit assessments on whether the REMS plan is meeting its goals or requires modifications [5]. In order to ensure the continuing adequacy of REMS plans, FDA assesses each plan on a periodic basis [5]. FDA can make modifications to strengthen a REMS when needed, eliminate elements that have proven to be overly restrictive, or release a REMS altogether [12].

Additionally, the FDAAA provides FDA with enforcement mechanisms for REMS violations [11]. If a manufacturer fails to comply with the requirements of its approved REMS, the drug may be deemed misbranded and removed from the market, and any responsible party may be subject to civil monetary penalties ranging from $250,000 to $10,000,000 [13, 14].

In 2012, Congress reaffirmed the importance of REMS by enacting the Food and Drug Administration and Safety Innovation Act (FDASIA) [15]. FDASIA required FDA to measure the effectiveness of REMS, continue to develop techniques to standardize REMS, and integrate REMS into the existing and evolving health care system [15].

#### FDA’s use of REMS has changed with experience

Since FDA implemented the REMS program in 2008, the agency has evolved in its use of REMS safeguards. Recognizing that REMS drugs represent a unique set of important medicines, FDA decided to streamline its REMS authority to concentrate on mitigating the risks of only the most potentially dangerous drugs. Therefore, in 2011, the agency began to release 145 less potentially toxic drugs from their REMS requirements out of the 222 REMS programs originally authorized [16]. As of October 2015, only 72 products have unique REMS programs in place, while six classes of products share REMS [17]. At the same time, just 12 of the 107 new medicines approved since 2012 required an authorized REMS program. This illustrates that the agency authorizes REMS only when necessary to protect patients from potentially severe adverse events.

#### Elements to assure safe use & restricted distribution

Given that REMS are intended to address the safety concerns regarding a specific medication, REMS elements are commensurate with the severity of risk [5]. To limit undue burdens on patient access, REMS programs must be compatible with established distribution, procurement, and dispensing systems for drugs and conform to plans for other drugs with comparable serious risks.

In situations where medicines have serious risks, FDA may require Elements To Assure Safe Use (ETASU), which are highly stringent safety precautions as part of the REMS initiative. Limited to situations where FDA determines that, due to a medicine’s “inherent toxicity or potential harmfulness,” the medication can only be approved with rigorous controls to ensure a serious or fatal risk can be avoided by proper use, these carefully planned safety protocols are the most extensive REMS elements [18]. As such, ETASU provide an avenue for patients with serious diseases and debilitating conditions to be treated effectively with drugs and biologics that would otherwise not be on the market.

Specifically, ETASU elements may require any or all of the following:

- That prescribers have specific training, experience, or special certifications;
- Pharmacies, practitioners, or health care settings dispensing the drug be specially certified;
- The drug be dispensed only in certain health care settings (e.g., hospitals);
- The drug be dispensed with evidence of safe use conditions, such as laboratory test results;
- Each patient using the drug be subject to monitoring; and
- Each patient using the drug be enrolled in a registry [5].

FDA will only approve a medication with ETASU if, due to an “inherent toxicity or potential harmfulness,” ETASU can ensure a serious or fatal risk can be avoided by proper use. This determination requires an assessment that other elements of REMS, such as a communication plan, are not sufficient, thus demonstrating that an FDA-approved medication necessitating REMS with ETASU is not issued lightly [5, 14].

Of the REMS drugs with ETASU on the market, a small number require restricted distribution systems to ensure that a drug or biologic known to cause birth defects, organ damage, and other serious or life-threating events is only acquired and used by patients under carefully controlled conditions. These systems may require physician qualification and registration, pharmacist distribution limitations, patients enrolling in a registry, and patients undergoing routine testing, such as women having a monthly pregnancy test [5, 19]. By restricting distribution to certified prescribers and pharmacists, it is more likely that only clinicians with a clear understanding of proper usage of the drug will prescribe it, thereby mitigating known safety risks [20].

Given that risk distribution programs must control the use of a drug or biologic from beginning to end, REMS with restricted distribution often differ in how they are prescribed, tracked, and controlled to ensure the safe return and disposal of unused quantities [1]. For example, a restricted distribution system may include some form of an access program in which wholesalers and distributors must enroll and commit to distributing only to authorized pharmacies participating in prescription tracking [21]. In this case, enrolled pharmacies are kept in databases, and authorized distributors and wholesalers are required to check the database to confirm that the pharmacy is an authorized entity to obtain the medication [21]. The drug manufacturer uses the database to monitor and evaluate the implementation of the access program requirements [21].

#### Samples for bioequivalence testing

For FDA to approve a generic form of a branded drug, manufacturers must submit an abbreviated new drug application (ANDA) demonstrating that the generic version is “pharmaceutically equivalent” to the innovator drug – meaning the drug contains the same active ingredient at the same strength, in the same dosage form (e.g., pill, capsule), and has the same route of administration.

Additionally, the manufacturer must establish bioequivalence, meaning that the generic version works in the body the same way, and is as safe and effective as the branded drug [14, 22]. FDA requires minimal trials involving human subjects that show the generic version performs in the same manner as the original innovator compound. One frequently used method is to measure the time it takes the generic drug to reach the bloodstream. If the generic version delivers the same amount of active ingredients into a patient’s bloodstream in the same amount of time as the original medicine, FDA determines it to be bioequivalent [23].

In order to conduct bioequivalence testing, generic manufacturers must obtain samples of the branded product to conduct comparison studies [22]. In most situations, generic companies are able to purchase brand drug samples from a wholesaler or distributor on the open market [24]. The problem comes in the rare situations when drugs are subject to REMS with ETASU and controlled through restricted distribution systems. In this instance, generic manufacturers are not authorized entities and approved wholesalers and distributors would violate of the terms of the REMS program if they were to sell samples to an unauthorized party.

Compounding the problem, brand manufacturers could violate their own REMS program if they shared samples with generic manufacturers for bioequivalence testing and face civil penalties [14]. In response to this problem, FDA issued *Draft Guidance: How to Obtain a Letter from the FDA Stating that Bioequivalence Study Protocols Contain Safety Protections Comparable to Applicable REMS for RLD* (“Draft Guidance”) in December 2014 [22]. The *Draft Guidance* explains how generic manufacturers can obtain samples of REMS drugs from the branded manufacturer [22]. It states that if the brand drug is subject to REMS with ETASU, a generic manufacturer can request a letter from FDA clarifying that the brand manufacturer will not violate its product’s REMS by providing drug samples to a generic manufacturer for bioequivalence testing [22].

To obtain this letter from FDA, the generic manufacturer must prove to the agency that in conducting its bioequivalence tests, the company’s protocol will incorporate the elements of the brand drug’s labeling and specific ETASU requirements [22]. In addition, there are specific responsibilities and liabilities generic manufacturers must accept if there is an adverse event. Through this process, nearly a dozen medicines subject to REMS have gone generic, including nine that are subject to stringent ETASU provisions.

#### Restricted distribution as required by REMS versus restricted distribution without REMS

It is important to note the distinction between medications that require restricted distribution as part of their REMS program and medications that require restricted distribution but do not have a REMS program. Medications with REMS have known or potential serious safety risks, so restricted distribution can ensure that these medications are only distributed to parties that have agreed to comply with all of the REMS requirements. In contrast, medications without REMS may lack such strong justifications.

In some outlier cases, bad actors may use restricted distribution for a medication that does not require REMS as an anti-competition mechanism to drive up prices. For example, Turing Pharmaceuticals recently imposed a restricted distribution system for phyrimethamine, even though the medication had no REMS program and had a well-characterized safety profile [25]. Turing then increased the price of the medication by 5000 % [25]. In this case, the adoption of the restricted distribution system did not have any medical justification and was only used to stave off competition.

## Methods

This article presents a study of REMS and legislation introduced to reform the REMS program in the United States. The analysis draws on diverse sources, including U.S. legislation, statutes, case law, government agency policies and guidelines, scholarly articles, and news stories published between January 1, 2004 and December 1, 2015.

This study describes the historical development of the REMS program, the REMS approval process, and enforcement mechanisms. The author provides legal and policy analysis to identify new challenges to REMS that may create unjustified risks to patient safety. The author then uses that analysis to develop and propose recommendations to preserve ETASU, and restricted distribution in particular, as a component of REMS.

## Results and Discussion

### New challenges to REMS are based on inaccurate information and create unjustified risks to patient safety

Despite the limited number of branded medicines approved with REMS requirements, critics have sought to weaken these safeguards, arguing that brand manufacturers use REMS controls, and especially restricted distribution systems, to prevent generic manufacturers from obtaining the product samples needed to conduct bioequivalence testing. Besides citing the difficulty obtaining samples of drugs subject to restricted distribution systems through customary distribution channels, the generic drug industry also alleges that innovator companies refuse to sell samples to generic marketers, thereby precluding them from satisfying FDA-approval requirements [26].

Citing these complaints, the generic drug industry has pressed for legislation to force brand manufacturers to sell samples of products subject to REMS with restricted distribution systems, and absolve generic companies from liability should a generic drug lead to serious injury, birth defects, or death. The generic manufacturer industry’s allies focus on the potential economic savings to patients from increased access to generic medicines and overlook the importance of REMS in ensuring the safe use of the small number of medicines that carry known safety risks when not carefully controlled.

### Dispelling falsehoods from interested parties

Today, millions of Americans with serious diseases (e.g., cancers, Crohn's disease, bowel disorders, chronic obstructive pulmonary disease, HIV, lung conditions, multiple sclerosis, seizures, and schizophrenia) and rare disorders are treated effectively with medications that may never have been available without REMS programs to ensure their safe use. However, eight years have passed since Congress authorized the REMS program and the sense of urgency that led to these requirements has been replaced by misperceptions about the need for REMS controls. Therefore, providing policy makers with up-to-date facts about the REMS program is critically important to correct inaccurate statements that are at the heart of the current REMS debate. Below summarizes specific allegations about the use of REMS programs and counterarguments.

#### Falsehood one: restricted distribution is a pretext

To make the case that forced sale legislation is needed, the Generic Pharmaceutical Association (GPhA) commissioned Matrix Global Advisors (MGA), an economic policy consulting firm, to prepare the report entitled *Lost Prescription Drug Savings from Use of REMS Programs to Delay Generic Market Entry* [27]. Issued in July 2014, the report contends that branded drug manufacturers use REMS safeguards as a pretext to deny generic manufacturers access to brand drug samples [26]. However, there are significant questions regarding the methodology used to reach this conclusion.

Even without an examination of the report’s methodology, readily available data from FDA dispels the conclusions of the MGA report. Explaining the purpose and intent of the REMS program, FDA states that certain drugs carrying known, serious risks can only be approved if the pharmaceutical manufacturer agrees to implement REMS controls with ETASU, such as restricted distribution systems, due to the drug’s inherent toxicity or potential harmfulness [5]. The agency defines a “serious risk” as one that results in or places the patient in danger of death, hospitalization, incapacitation or disruption of the ability to conduct normal life function, congenital anomaly, or a birth defect [5]. One such example is vigabatrin, an anti-seizure drug known to cause permanent blindness and suicidal tendencies if the medicine is not used by patients under carefully controlled conditions [28]. Through restricted distribution, FDA was able to build in safeguards to the REMS program so patients with seizures could be treated with vigabatrin [5].

However, critics discount the need for these precautions and overlook the potential for drugs carrying known serious risks to be diverted, by theft or otherwise, and distributed with no safeguards in place. This is a growing concern for both regulators and the public health community in light of a growing black market for drugs that are obtained through employee pilfering, pharmacy robberies, theft from patients, and other means, which are then sold at a profit to unsuspecting distributors, hospitals, pharmacists, physicians, and consumers.

To appreciate the consequences, consider the potential for harm if ambrisentan, a pulmonary hypertension drug, were diverted. Because ambrisentan can cause serious birth defects if taken by a pregnant woman, FDA requires the manufacturer to operate a restricted distribution system under REMS that entails a monthly pregnancy test for women of childbearing age in addition to other safeguards [29]. Without these controls, diverted ambrisentan could lead to newborns with severe deformities.

Critics further ignore the legal precedent for liability challenges against branded manufacturers if medication errors are linked to generic forms of high-risk drugs and biologics [25]. Because generic drugs are copies of branded medicines, courts have held that generic manufacturers are not liable for the adverse events caused by the generic drug; instead, the brand manufacturer is liable^1^ [30–32]. Moreover, if a generic medication yields a significant increase in adverse events, FDA could also require additional REMS elements of the innovator company or withdraw the drug from the market altogether [25]. Such adverse events associated with the generic product could also hurt the brand firm’s reputation [25].

In light of these challenges, FDA has stated that the current liability doctrine “alters incentives for generic drug manufacturers to comply with current requirements to conduct robust post-marketing, surveillance, evaluation, and reporting, and to ensure that the labeling for their drugs is accurate and up-to-date [29].” In other words, this liability rule provides further disincentive for generic manufacturers to bear the expense of monitoring the safety of their products and implementing systems to prevent adverse events.

#### Falsehood two: a large number of generic medications are impacted by restricted distribution

REMS programs are rare and only authorized when necessary to protect patients from potentially severe adverse events. This is especially true for medicines approved with REMS programs requiring ETASU. Approximately half the medicines with authorized REMS programs are subject to ETASU and an even smaller number require restricted distribution systems.

However, this fact is clouded by inaccurate projections in the MGA report that “nearly 40 % of new FDA approvals are subject to REMS, and the percentage of REMS programs that require distribution restrictions has increased dramatically in the last several years [26].” To produce this estimate, MGA applied pre-2011 FDA data, which is now completely out of date, rendering the findings both misleading and inaccurate. In 2011, FDA streamlined the REMS program and released 145 of the 222 medicines originally approved with REMS requirements, which the MGA report does not take into account. In taking this action, FDA was careful to ensure that drugs requiring restricted distribution systems meet specific FDAAA requirements so the REMS elements commensurate with the specific risks posed by the drug and are not unduly burdensome to patient access [5]. As a result, only a small number of medications with REMS have restricted distribution systems.

This process was triggered in November 2011, when FDA published guidance to allow drug manufacturers to request removal of their REMS requirements when the only element is a medication guide [33]. By early 2012, the agency had released 96 drugs with medication guide-only REMS and removed medication guides as an element from 14 other REMS programs that require a communications plan [1].

In taking these steps, FDA was careful to ensure that drugs requiring restricted distribution systems meet specific FDAAA requirements so the REMS elements commensurate with the specific risks posed by the drug and are not unduly burdensome to patient access [5]. The result is only a small number of medications are now marketed through restricted distribution systems.

Also of note, FDA continues to reevaluate medicines with REMS designations, modifying or releasing REMS requirements when agency officials deem them no longer necessary [34]. For instance, in February 2011, FDA removed the restricted distribution requirement for two drugs – romiplostim injection and eltrombopag tablets – after determining some of the adverse events associated with these medications were part of the natural history of the illnesses treated by these drugs [35]. In November 2011, FDA published guidance to allow drug manufacturers to request removal of their REMS requirements when the only element is a medication guide [32]. Thus, by early 2012, FDA had released 96 drugs with medication guide-only REMS and removed medication guides as an element from 14 other REMS programs that require a communications plan [1].

As a result of FDA’s actions, the number of branded drugs that now have authorized unique REMS programs is not 40 % as MGA claims, but six percent – or 73 of the 1,161 FDA-approved brand drugs [36]. Of these 73 medicines, 36 have ETASU requirements (3 %), and 31 are subject to restricted distribution (2.5 %) [17]. Therefore, even as FDA works to increase the number of drug approvals, the chart below clearly shows that the number of new medicines requiring restricted distribution remains very low and has not changed significantly between 2010 and 2014.

**Table:** 

	2010 [17, 37]	2011 [17, 38]	2012 [17, 39]	2013 [17, 40]	2014 [17, 41]
# of new FDA-approved drugs	21	30	39	27	41
# of new FDA-approved drugs with REMS with restricted distribution	1	1	1	5	1
Percentage of new FDA-approved drugs with REMS with restricted distribution	4.7 %	3.3 %	2.5 %	18.5 %	2.4 %

#### Falsehood three: generic manufacturers have no access to samples

It is true that drugs requiring restricted distribution systems are more difficult for generic drug manufacturers to obtain due to controls over when and to whom the drug may be sold. However, the assertion in the MGA report that generic drug manufacturers have no access to medications for bioequivalence testing due to REMS with restricted distribution is not supported by the facts [26].

Based on current REMS safety protocols, nearly a dozen medicines subject to REMS requirements have generic counterparts on the market, including nine subject to stringent ETASU provisions. This includes generic versions of three branded testosterone gels and bupropion hydrochloride [42]. Similarly, Roxane Laboratories submitted an application for a generic version of alosetron hydrochloride, despite the branded drug being subject to REMS with restricted distribution [43].

Generic manufacturers have also been successful in testing and receiving FDA clearance to market five branded drug products that share a single REMS system [44]. Accordingly, generic versions are now available for buprenorphine transmucosal products to treat opioid dependence and four other medicines subject to restricted distribution requirements: isotretinoin to treat acne; the immunosuppressant drug mycophenolate to prevent organ rejection after transplant; the diabetes drug rosiglitazone; and transmucosal immediate release fentanyl, used to manage breakthrough pain in adults with cancer.

These approvals are the results of established procedures whereby FDA permits a branded manufacturer to sell quantities of drugs subject to restricted distribution to a generic manufacturer for bioequivalence testing. The requirement in such instances is that generic manufacturers make reasonable assurances to FDA and the branded manufacturer that the drug will be handled, dispensed, and administered safely. In addition, there are certain responsibilities and liabilities generic manufacturers must accept if there is an adverse event.

#### Falsehood four: lack of samples has resulted in billions of dollars in avoidable costs

The MGA report projected $5.4 billion a year in U.S. health care savings if generic versions of select branded drugs “whose market entry is currently delayed by misuse of REMS or other restricted access [p]rograms” are allowed to come to market [26]. Yet, this conclusion is conjecture and based on questionable methodology.

To make its projection, MGA researchers surveyed some of the GPhA member companies (not all participated in the survey) who identified “brand drugs with REMS *or other* restricted access programs reportedly used to prevent generic manufacturers from accessing drug samples [26].” There is no explanation in the MGA report of the criteria used by the survey respondents, which is problematic. Based on these findings, MGA analyzed 40 unspecified drugs “with programs to block generic access,” of which only 16 (40 %) actually have REMS requirements [26]. The other 24 drugs, according to the report, use “non-REMS restrictions to block access,” although the report does not describe the restrictions and how they are used [26].

Compounding this flawed analysis, MGA attributes over 50 % of sales for these 40 brand drugs to four products; yet the report does not state whether these medicines are actually subject to REMS controls [26]. In calculating the “economic cost of REMS misuse,” MGA also made the assumption that FDA will approve generic drug applications for all 40 innovator medicines; however, there is no guarantee this will occur. For instance, FDA rejected 18 % of all generic drug applications in 2010 and 15.5 % in 2011 [45]. In 2012, FDA rejected 100 applications (9.4 %), and 40 of those applications were rejected due to serious bioequivalence deficiencies despite access to samples [44].

Lastly – and of critical importance when approving medicines known to carry high risks – MGA researchers did not take into account the costs that would arise if generic manufacturers did not implement the same rigorous REMS safeguards as the innovator company and patients experienced adverse events. Unfortunately, the reality is these costs could be substantial, including the possibility that both the innovator drug and the generic version could be removed from the market.

### Forced sale is not permissible under current law

Although REMS has become an essential tool to advance patient safety, proposed legislation introduced in the 114th Congress would undermine the most important purpose of REMS: Ensuring drugs with potentially dangerous risks are safely accessible only to the appropriate people. Specifically, the Fair Access to Safe and Timely (FAST) Generics Act of 2015, introduced on June 18, 2015, would require the “forced sale” of drugs subject to REMS requirements as samples for bioequivalence testing [46].

From a patient safety perspective, the implications are significant: If the forced sale provision were to be enacted, the changes would undermine necessary drug safety precautions and create disincentives for the future development and marketing of higher-risk drugs, especially to treat rare disorders, due to liability concerns. Moreover, the proposed legislation raises serious legal challenges. While FDA has statutory authority to regulate REMS and require restricted distribution systems, no legal authority currently exists to mandate sales to generic companies [24, 47].

In light of these concerns, when Congress passed FDAAA, legislators excluded a provision requiring FDA to mandate that branded manufacturers provide samples to generic manufacturers for bioequivalence testing. Similarly, in 2012, lawmakers passed the Food and Drug Administration Safety and Innovation Act (FDSIA), which excluded a forced sale provision because Congress did not want it to lead" to FDA forcing drug sales between brand and generic manufacturers” [48]. Then in 2014, the FAST Generics Act was introduced for the first time, which specifically focused on the forced sale of branded medicines requiring restricted distribution systems to ensure safe use to generic manufacturers [45]. The bill died in committee, but was reintroduced in June 2015 [47].

Reinforcing these actions, FDA has publicly stated its position on requiring the forced sale of branded drugs to generic manufacturers for bioequivalence testing. Responding to a Citizen Petition filed by a generic manufacturer, the agency rejected the request to establish procedures to “prevent companies from using REMS to block or delay generic competition” [49], stating that market competition issues are best addressed by the Federal Trade Commission (FTC) [50].

Moreover, through its *Draft Guidance on Safety Letters* issued in December 2014, FDA made clear it will not compel branded manufacturers to sell samples of medicines subject to REMS controls. However, the guidance clarifies the process by which a generic manufacturer can obtain a letter from FDA stating the safety protections proposed for the bioequivalence study are comparable to the innovator company’s REMS program. This letter, in effect, permits the sale of branded drug samples for bioequivalence testing [51].

### The safety implications of legislative proposals to force the sale of branded drugs subject to REMS requirements are significant

By forcing the sale of medicines subject to REMS with restricted distribution for bioequivalence testing, legislative proposals like the FAST Generics Act present significant safety risks that should not be overlooked. This includes the provision forcing innovators to sell drugs subject to REMS with ETASU without specific requirements for generic manufacturers to implement the same rigorous safeguards to ensure safe use [45]. The proposed legislation would also nullify the current process by which FDA permits an innovator company to sell samples of a REMS drug for bioequivalence testing and expose the brand manufacturer to liability without giving the manufacturer the opportunity to mitigate risks or negotiate indemnity.

### Restricted distribution systems controlling the use of high risk medicines would be weakened

Intended to remove what the generic drug industry perceives as anticompetitive obstacles to faster approval of more generic medicines, the FAST Generics Act contains language that will significantly weaken existing restricted distribution systems for medicines known to carry serious risks. Specifically, the bill reads that brand manufacturers may not “adopt, impose, or enforce any condition relating to the sale, resale, or distribution of the covered product, including any condition adopted, imposed, or enforced as an aspect of a [REMS] that restricts or has the effect of restricting the supply of [the brand product] to an eligible product developer for development or testing purposes [45].”

Moreover, if the FAST Generics Act were enacted, restricted distribution systems that dispense high-risk medicines only to certified pharmacies, hospitals, or prescribers would be in violation of federal law [45]. The bill states that REMS cannot prohibit or restrict the supply of a drug for development and testing purposes by anyone seeking to prepare a drug marketing application [45]. Therefore, instead of allowing FDA to determine when a restricted distribution system is required for the safe use of a medicine, the proposed legislation would remove FDA from the process. The only exception under the FAST Generics Act would be for FDA to create a carve-out in the REMS plan that would allow for distribution to eligible product developers.

### The sale of medicines known to carry high risks may not be limited to generic manufacturers

The bill broadly and ambiguously defines an “eligible product developer” as “a person that seeks to develop a [generic drug application] [45].” By this definition, any individual or business could claim to be an eligible product developer, including an entity with a history of failing to follow safety protocols or producing counterfeit or adulterated medications, as long as the business or individual intends to file a generic drug application with FDA.

Compounding the problem, language in the FAST Generics Act states that a REMS plan may not prohibit or restrict the brand manufacturer, authorized wholesaler, or specialty distributor from providing an eligible product developer with a supply of product for development and testing purposes [45]. If this provision were to go into effect, wholesalers and distributors would be able to sell large quantities of drug samples essentially to anyone in the stream of commerce, increasing the likelihood of diversion and defeating the purpose of ETASU.

To address this serious problem, the bill’s sponsors added provisions authorizing FDA to prohibit, limit, or otherwise suspend the sale of a brand drug subject to REMS requirements to a “product developer” when the agency determines there would be an imminent hazard to the public health [45]. This provision would allow FDA to take action when it is aware that a “product developer” failed safety protocols in the past or operates on the black market. However, the proposed legislation would allow a wholesaler or distributor to sell samples without revealing the identity of the purchaser to the brand manufacturer, meaning there may be situations in which these samples get into the hands of a bad actor, yet neither FDA nor the brand manufacturer would have any knowledge of the transfer [45].

### Expanded use of shared REMS systems may be impeded

FDA encourages greater use of shared system REMS when a number of generic versions of a medication require the same ETASU. This is because each shared REMS program provides a single web portal to access medication guides, prescribing information, and other information, making it easier to simplify patient registries and prescriber and pharmacist education platforms.

The FAST Generics Act may have the unintended consequence of hampering expanded use of the single, shared REMS system. While the bill states no brand manufacturer “shall take any step that impedes the prompt development of a single, shared system of [ETASU],” the proposed legislation fails to define what constitutes a “step to impede prompt development [45].” As a result, the time it normally takes for the innovator company and generic manufacturer to negotiate each party’s responsibilities to implement a shared REMS system could be challenged as “steps to impede prompt development.” Moreover, because generic manufacturers are not liable for the adverse events occurring from use of their medications under the current liability doctrine, generic companies will have no incentive to engage in good faith negotiations with the innovator on operating a single, shared REMS system.

### The proposed legislation would create a lax system where potentially dangerous drugs may get into the wrong hands

Acting under the belief that REMS programs block generic drug development, the FAST Generics Act would make it unlawful for branded manufacturers to refuse to sell samples of products subject to REMS controls for any reason. To that end, the bill gives FDA new authority to compel the branded manufacturer and its wholesalers or distributors to provide product samples for bioequivalence testing and place safety conditions on the generic manufacturer [45]. However, the bill gives FDA a deadline of 60 days to authorize the forced sale, and if the agency does not meet this deadline, authorization is automatic [45]. As a practical matter, 60 days in a very limited time under any circumstances, let alone one that may affect the public health by exposing patients to drugs with serious, known risks.

Regardless of the time limitations, the consequences for branded manufacturers would be significant. Specifically, an innovator company that fails to comply with the Act would be treated as if the company violated its own REMS program [45]. The result could be civil penalties imposed as a REMS violation and legal action taken by the company or individual requesting samples seeking injunctive relief and treble damages [45].

Complicating the situation, the proposed legislation gives generic manufacturers the right to obtain large quantities of an innovator drug from a wholesaler or distributor while preventing the brand manufacturer from learning the identity of the company or individual receiving quantities of the innovator’s drug [45]. Under the bill, the wholesaler or distributor would only be allowed to inform a brand manufacturer that a request for product samples had been received. Therefore, brand manufacturers will have no way to comply with REMS requirements to monitor and track the use of high risk medicines while wholesalers and distributors will feel free to do business with any party that offers adequate consideration for drug samples under the guise of an eligible product developer.

### The proposed legislation would weaken existing systems to monitor and track the use of high risk medicines

In addition to contravening existing intellectual property and antitrust laws, which state that a manufacturer is free “to exercise his own independent discretion as to parties with whom we will deal,” the proposed FAST Generics Act will create a detrimental environment for the future development and marketing of higher-risk drugs, especially to treat rare disorders, due to liability concerns.

Although the bill contains a provision exempting the brand manufacturer from liability if individuals are harmed by the failure of the generic drug developer to implement REMS safety protocols, in reality, it would be difficult, if not impossible, to assign responsibility for harm, resulting in both reputational and financial damage to the brand manufacturer [45]. Moreover, the provision does not address any liability the brand manufacturer may face after the bioequivalence testing period is over, especially because the bill opens up the chain of distribution with the risk that potentially dangerous drugs will get into the wrong hands and cause serious adverse effects. In such a case, the branded manufacturer may face liability for harm suffered by patients, harm to its reputation, and the possibility that its drug will be taken off the market all because existing drug safety precautions under REMS were not in effect.

The future supply of generic medicines depends on policies that encourage, not discourage, the development of new therapies. For this reason, policies that would undermine long-standing intellectual property protections by forcing the sale of branded drug products from one company to another are not in the public interest.

Furthermore, there is legal precedent for liability challenges against innovator companies if medication errors are linked to generic forms of high-risk drugs and biologics [52]. Even though an increasing number of courts are declining to hold branded manufacturers liable for injuries allegedly caused by generic-equivalent drugs, legislative proposals now being considered could open the door to a wave of liability suits because generic developers are not required to follow the same rigorous controls to ensure the safe use of high-risk medicines as innovator companies. If this were to happen, there would be a chilling effect on the development and marketing of new drugs subject to REMS restrictions in the future.

### Recommendations: FDA must preserve ETASU as a component of REMS

Today, the REMS program envisioned by Congress and implemented by FDA has become an essential tool to advance patient safety, protect public health, and provide access to innovative medicines that would otherwise not be available. In the years that the REMS program has been in effect, it has evolved to mitigate the risks of only the most potentially dangerous drugs. As a result, REMS programs are rare and only authorized by FDA to prevent life-threatening complications, birth defects, severe allergic reactions, and infections resulting from the inappropriate use or handling of higher risk drugs.

Yet, legislative proposals under consideration in the 114th Congress to force the sale of medicines carrying serious risks to generic marketers for bioequivalence testing could significantly weaken the drug safety protections REMS makes possible. Although the goal of speeding the development of less expensive generic medicines is laudable, bills such as the FAST Generics Act would gut existing safety measures and increase the risk to public health by making it easier for potentially dangerous drugs to get into the wrong hands by:

- Strongly undermining existing restricted distribution systems for medicines known to carry known serious risks.
- Defining an eligible product developer able to obtain high-risk branded medicines as any person or entity that seeks to develop a generic drug.
- Allowing wholesalers and distributors to sell large quantities of drug samples without safety precautions.
- Preventing brand manufacturers from learning the identity of the company or individual receiving the company’s drug and, therefore, impeding the manufacturer’s ability to monitor and track the use of this potentially dangerous medicine.
- Allowing FDA limited time to authorize the forced sale of drug samples to “eligible product developers.” If the agency is not able to act in time, authorization would be automatic.

Guided by the need to prevent harmful exposure to medicines that can cause harmful birth defects, organ damage, and even death when not handled and administered with the utmost care, policy makers must exercise great caution when considering changes to the current REMS program. Accordingly, policies that would allow the forced sale of drugs known to carry high risks without required safeguards to ensure these medicines are handled and administered safely are not in the public interest and should not be implemented.

However, guarding patient safety and increasing generic drug development are mutually exclusive. Therefore, policy makers must take steps to preserve REMS safeguards while encouraging the development of policies that make it easier for brand manufacturers to negotiate with generic companies based on certain agreed upon responsibilities and liabilities under FDA’s continued oversight. To achieve these important goals, a policy framework should:

- Recognize the need for REMS programs to ensure continued patient access to innovative, higher risk medicines.
- Preserve REMS programs using ETASU, including restricted distribution systems, when necessary to mitigate the risks of potentially dangerous drugs.
- Hold all drug manufacturers – branded and generic – to the same safety requirements when designing and conducting drug studies, including bioequivalence testing.
- Allow brand and generic manufacturers to negotiate the terms under which product samples are provided for bioequivalence testing.
- Preserve the intellectual property rights of innovator companies when implementing REMS policies.

## Conclusion

REMS are necessary to make medications with known severe risks available to certain patient populations for whom treatment may not be available otherwise. FDA only requires REMS with restricted distribution for medications that would otherwise not be allowed on the market due to their risks, and only a small number of medications are subject to REMS with restricted distribution. In order to ensure proper safety measures are preserved and medications with known risks are not diverted to parties who will not follow safety requirements, legislation should not be passed to require a forced sale of drugs subject to REMS with restricted distribution for bioequivalence testing purposes. Generic manufacturers must be held to the same REMS safety standards as brand manufacturers. Systems currently in place adequately balance risk and safety.

## Footnotes

1 Note that the FDA proposed a rule to allow generic manufacturers to update product labeling to reflect newly acquired safety information even if the updated language differs from the innovator drug, and distribute the product with differing labeling upon submission to the FDA of a “changes being effected” (CBE-o) supplement. Under the rule, it is possible that a generic manufacturer may face liability for adverse events that occur while the generic drug has different labeling from its innovator drug. The FDA is expected to issue the final rule in September 2015.
